# The Clinical Utility of Repeating Magnetic Resonance Imaging (MRI) Scans Within 12 Months in the Management of Lumbosacral Degenerative Disc Disease

**DOI:** 10.7759/cureus.53100

**Published:** 2024-01-28

**Authors:** Muhammad Ashhad Noor, Mohammad Al-Ashqar, Ahmad Abul, James Grayston, Sohail Nisar, Peter R Loughenbury, Graham Radcliffe

**Affiliations:** 1 Department of Medicine and Surgery, Manchester University National Health Service (NHS) Foundation Trust, Manchester, GBR; 2 Department of Trauma and Orthopaedics, Yorkshire and Humber Deanery, Leeds, GBR; 3 College of Medicine, University of Leeds, Leeds, GBR; 4 Department of Trauma and Orthopaedics, University Hospital Crosshouse, Kilmarnock, GBR; 5 Department of Trauma and Orthopaedics, Bradford Royal Infirmary, Bradford, GBR; 6 Department of Spinal Surgery, Leeds Teaching Hospitals National Health Service (NHS) Trust, Leeds, GBR

**Keywords:** mri, surgical intervention, repeat scanning, clinical management, radiological change, lumbosacral degenerative disc disease

## Abstract

Purpose: Magnetic resonance imaging (MRI) is the gold standard investigation for lumbosacral degenerative disc disease. However, there is controversy regarding the clinical value of repeating an MRI scan within 12 months when a patient presents with recurring or changing symptoms. This study measures rates of radiological change in a real-world cohort to guide clinicians when deciding to repeat a scan.

Methods: All patients over a 10-year window in one general hospital who underwent two lumbosacral MRI scans for degenerative disc disease within 12 months of each other were included in the study. All MRI reports were manually reviewed. The level of main vertebral pathology was recorded, along with the location of a disc prolapse. Time intervals between the two scans were calculated, and these were collated into 30-day intervals for analysis. The repeat scans were categorized into three groups: no change, radiological improvement, and radiological deterioration. Patients who had clinically significant deterioration in the form of cauda equina compression on MRI scans were recorded.

Findings: Four hundred and eighty-one patients were included for analysis. Three hundred and ninety (81%) showed no change in MRI findings, 18 (3.7%) had improvements in their repeat scans, and 73 (15.3%) demonstrated deterioration in their repeat scans. Of the 73 patients with radiological deterioration, three patients (0.62% of the total) required urgent surgical intervention for cauda equina syndrome (CES).

Conclusions: Though there is no alternative to detailed clinical assessment in determining whether a repeat MRI scan is indicated, the findings demonstrate that repeating MRI within 12 months for patients with lumbosacral degenerative disc disease has a low chance of altering the management plan. Over the 10-year period, only three patients required an urgent change to their clinical management. We believe this data can help guide clinical decision-making when considering a repeat scan.

## Introduction

Lumbosacral degenerative disc disease is a prevalent condition characterized by a wide spectrum of clinical presentations, ranging from mild low back pain or sciatica to severe cauda equina syndrome (CES) [1]. Conservative management, including analgesia and physiotherapy, is the initial approach for most patients with low back pain or sciatica [2]. However, in cases where conservative therapy fails or there is suspicion of neurological compromise, magnetic resonance imaging (MRI) is often employed as the gold standard investigation to identify patients who may benefit from surgical intervention [3]. This non-invasive imaging modality provides invaluable visualizations of the soft tissue anatomy, allowing for the accurate assessment of disc prolapse and the extent of nerve or spinal compression in lumbosacral degenerative disc disease [3].

As the prevalence of degenerative disc disease in the UK population exceeds the capacity for MRI scanning, the National Institute for Health and Care Excellence (NICE) has produced guidelines detailing the indications for MRI scanning [4]. While this guidance is particularly relevant for initial presentations, the question of whether MRI should be repeated in cases where patients present with new or exacerbated symptoms after a previously reassuring MRI remains equivocal [5]. Consequently, clinical practice demonstrates considerable variability, as the decision to pursue repeat imaging is left to the discretion of individual healthcare practitioners [3].

The aim of this study is to determine how often clinically significant changes requiring surgery are detected when repeating spinal MRI within 12 months. This information will help clinicians make informed decisions and provide practical guidance to patients regarding the potential benefits of repeating an MRI scan within a 12-month period.

## Materials and methods

Study design and population

This retrospective study examines patients who underwent two consecutive lumbosacral spine MRI scans within a 12-month timeframe. The data were obtained from the radiology department of Bradford Royal Infirmary, a prominent teaching hospital in the United Kingdom, and encompassed a 10-year period from 2009 to 2019. Retrospective data collection and analysis spanned from January 1, 2020, to June 1, 2020.

Ethical approval

Since the study was retrospective in design, the collection and review of patient data had no impact on patient outcomes or treatments. Furthermore, the research involved completely anonymised data sets making it impossible to identify individuals from the provided records. Hence, the study was exempt from ethical approval.

Inclusion and exclusion criteria

**Figure 1:**
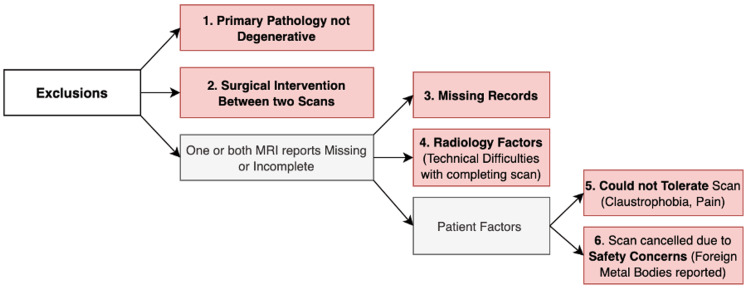
Flowchart illustrating the exclusion criteria

Included in the study were patients diagnosed with lumbosacral degenerative disc disease based on their initial MRI scan. All patients had previously received a reassuring MRI scan but re-presented within 12 months due to worsening symptoms, prompting a repeat MRI scan to reassess for the presence of CES. Therefore, patients who had CES identified on their initial scan and required neurosurgical intervention were excluded from the study. Additional exclusion criteria are detailed in Figure 1. 

Data collection

For each patient episode, the following demographic data were collected: hospital identification number, age at the time of initial MRI scan, date of initial MRI scan, radiology report of initial MRI scan, date of repeat MRI scan, and radiology report of repeat MRI scan. All episodes were coded for tracking purposes.

Both MRI reports for each patient episode were manually reviewed. The level of vertebral pathology was meticulously recorded, with cases of multi-level pathology noted as "Multiple" and cases with no pathology recorded as "N/A." The location of disc prolapse was documented as central, paracentral, or lateral recess. The grade of the most experienced reporting radiologist was also recorded. The time interval between the two MRI scans was calculated, and any changes in MRI findings were carefully documented as "Radiological Improvement," defined by a decrease in the size of existing disc prolapse on imaging, or "Radiological Deterioration," defined by a new disc prolapse or increase in the size of existing prolapse on imaging. In addition, we identified episodes of deterioration that demonstrated new-onset CES; these were recorded as "Clinically Significant" episodes since they prompted an urgent change to the management plan. Following the initial analysis, a comprehensive review of all records was conducted by MA and SN to ensure consistency and accuracy in the interpretation of MRI reports for data collection.

Data analysis

The primary outcome of interest in this study is the identification of new CES following a repeat MRI scan. The time intervals between the two MRI scans were systematically categorized into 30-day periods for comprehensive analysis. Subsequent analyses were conducted to explore potential associations between the location of disc prolapse in the initial MRI scan (i.e., central, paracentral, lateral recess, or N/A) and the outcomes observed.

## Results

**Figure 2:**
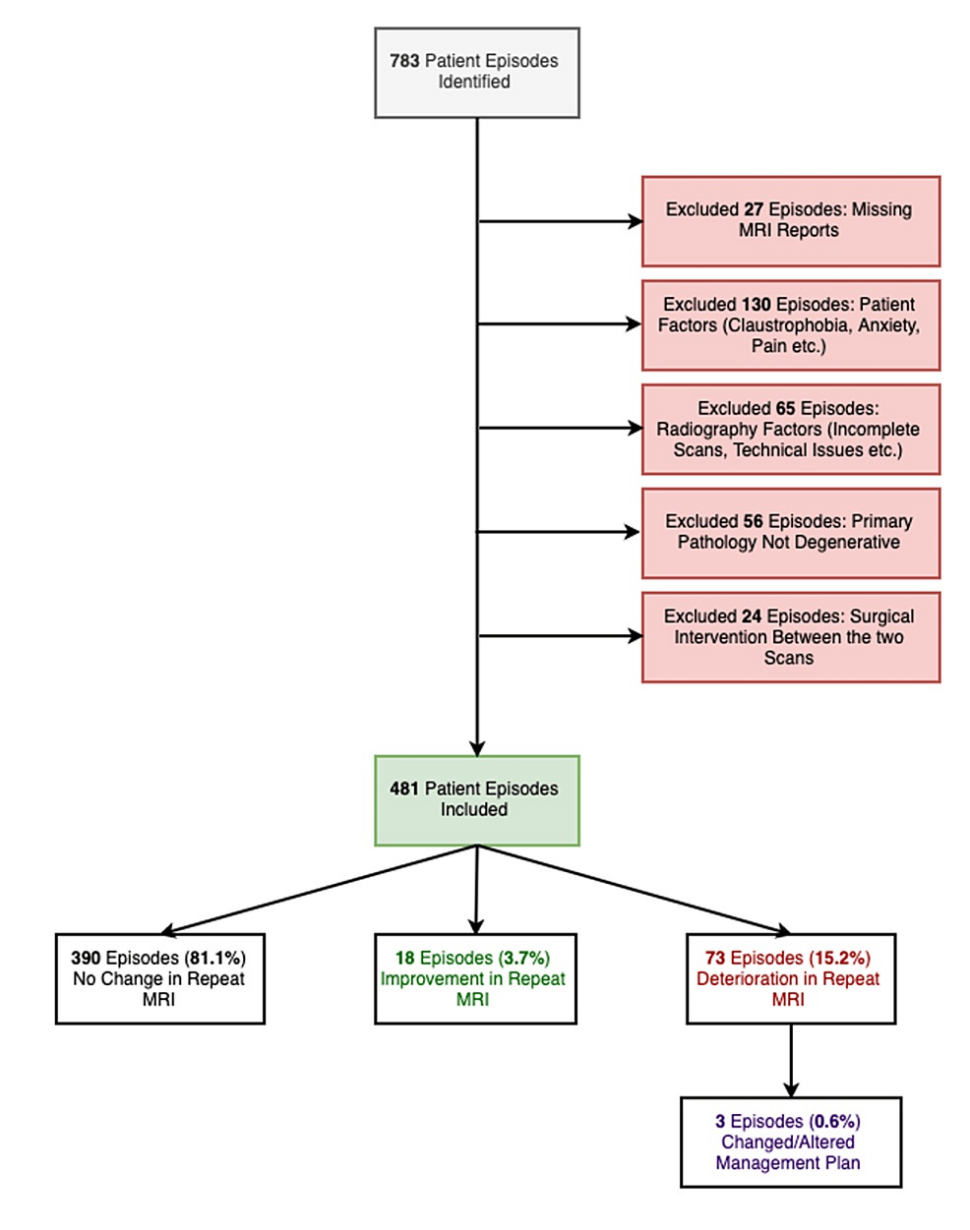
Flowchart illustrating study inclusion/exclusion criteria and cohort

A total of 783 patient episodes spanning a 10-year period from 2009 to 2019 were identified through comprehensive electronic searches. All MRI reports were reported or vetted by a consultant radiologist. Figure 2 provides detailed information on the 302 patient episodes that were excluded from the study according to predetermined exclusion criteria.

A total of 481 patients diagnosed with lumbosacral degenerative disc disease were included for analysis in this study. The mean age of the patients was 46 years, with a range from 14 to 94 years. Among the included cases, 221 patients had disc disease identified at multiple vertebral levels. The most frequently affected single level was L5/S1, which was observed in 134 patients, followed by L4/L5, which was identified in 89 patients, as outlined in Table 1.

**Table 1:** Level at which degenerative disc disease was found NA: not applicable

Level of degenerative disc disease	Number of patients
L5/S1	134
L4/L5	89
L3/L4	11
L2/L3	2
L1/L2	2
T12/L1	2
Multi-level	221
NA (degenerative changes without disc bulge)	20

In the analysis of reported MRI changes, it was found that most participants, comprising 390 individuals (81%), showed no significant alterations in their MRI findings between the initial and subsequent scans. On the other hand, 18 participants (3.7%) demonstrated improvements in their repeat scans. Meanwhile, 73 individuals (15.3%) experienced deteriorations in their repeat scans.

Out of the 73 patients who experienced radiological deterioration according to the MRI findings, a total of three patients, constituting 0.62% of the total cohort, had a new finding of CES, leading to an urgent change in their management plan in the form of urgent neurosurgical intervention. These cases included patient no. 4, aged 60 (presented on March 9, 2009), with a 260-day gap between scans and an initially reported paracentral disc position; patient no. 524, aged 41 (presented on August 21, 2016), with 124 days between scans and an initially reported central disc position; and patient no. 770, aged 39 (presented on October 22, 2018), with 90 days between scans and an initially reported central disc position.

What number of MRI scans have a radiological change in each time interval?

**Figure 3:**
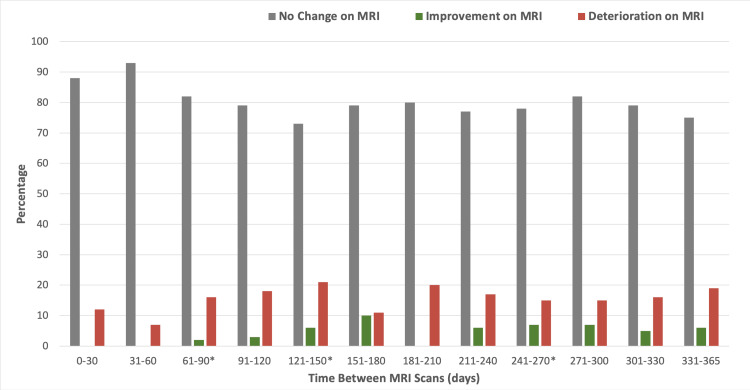
Bar chart illustrating rates of radiological change in MRI scans, according to time periods between scans MRI: magnetic resonance imaging

The time intervals between scans in this study were collated into 30-day intervals for analysis, as detailed in Table 2. Overall, 81% of patients demonstrated no change in MRI findings between scans, with the highest percentage of scans with no change observed in the intervals of 31-60 days (93%) and 121-150 days (73%). Notably, three episodes of clinically significant deterioration requiring surgical management were identified in distinct time intervals, 61-90 days, 121-150 days, and 241-270 days. These findings are presented in Figure 3, which further elucidates that there was no discernible correlation between the incidence of radiological changes in MRI findings and the time interval between scans.

**Table 2:** Rates of change in MRI according to time periods between scans: 30-day increments MRI: magnetic resonance imaging

Time between MRIs (days)	Total number of patients	Average age (years)	% no radiological change	% radiological deterioration	% clinically significant deterioration
0-30	57	47	87.7	12.3	0
31-60	57	47	93	7	0
61-90	44	45	81.8	15.9	2.3
91-120	34	48	79.4	17.6	0
121-150	33	44	72.7	21.2	3
151-180	28	46	78.6	10.7	0
181-210	25	42	80	20	0
211-240	35	48	77.1	17.1	0
241-270	40	50	77.5	15	2.5
271-300	33	48	81.8	15.2	0
301-330	43	43	79.1	16.3	0
331-365	52	47	75	19.2	0
Total	481	46	81.1	15.2	0.6

What is the incidence of radiological change in MRI by location of disc?

Central Disc Position

Based on the initial MRI scans, central disc pathology was identified in 307 cases, with 260 of these cases (84.7%) showing no change in disc position upon follow-up scans, while 39 cases (12.7%) demonstrated deterioration. Notably, two of these cases required surgical management due to clinically significant changes, as outlined in Table 3.

**Table 3:** Rates of change in MRI according to the location of the main disc N/A: not applicable (degenerative changes without disc bulge); MRI: magnetic resonance imaging

Location of the disc in the original MRI	Central	Paracentral	Lateral	N/A	All
Total number of patients	307	91	39	44	481
Average age (years)	47	43	51	46	46
Mean time between scans (days)	178	180	149	158	174
Number with no radiological change	260	58	30	42	390
% no radiological change	84.7	63.7	77	95.5	81.1
Number with radiological deterioration	39	26	7	1	73
% radiological deterioration	12.7	28.6	17.9	2.2	15.2
Number with clinically significant deterioration	2	1	0	0	3
% clinically significant deterioration	0.7	1.1	0	0	0.6

Paracentral Disc Position

Paracentral disc pathology was observed on the initial MRI scan in 91 cases, with 58 cases (63.7%) showing no change in disc position on subsequent scans, while 26 cases (28.6%) exhibited deterioration. One of these cases required surgical management due to clinically significant changes.

Lateral Recess Disc Position

For lateral recess disc pathology, there were 39 cases identified on the initial scan, with 30 cases (76.9%) showing no change in disc position on follow-up scans, while seven cases (17.9%) demonstrated deterioration. None of these cases were clinically significant and did not require surgical intervention.

Disc Position N/A

In cases where disc position was not applicable due to the absence of disc prolapse on the initial scan (44 cases), 42 cases (95.5%) showed no change on subsequent scans, while two cases showed changes, with one case (2.2%) demonstrating deterioration. Neither of these changes was clinically significant and did not require surgical intervention.

## Discussion

Our study findings highlight that repeat MRI within 12 months for patients with lumbosacral degenerative disc disease has a low probability of altering patient management. The overall likelihood of repeat MRI scans showing no change in radiological findings is approximately 81%, while the chance of demonstrating radiological deterioration is approximately 15%, and radiological improvement is approximately 4%. Importantly, over a 10-year period, only three patients (0.62%) demonstrated "clinically significant" deterioration, with MRI findings demonstrating CES. A recent systematic review by Hoeritzauer et al., which analysed 26 existing studies, determined an incidence of CES in 19% of symptomatic patients with lumbosacral degenerative disc disease [6]. When we consider our finding of only 0.62% incidence of CES in symptomatic patients who represent within a year, we determine that the probability of identifying CES in this cohort is approximately 30 times lower than for first-time presentations.

While central disc protrusions were the most common type observed in our cohort, our findings revealed that patients with paracentral disc protrusions were more likely to exhibit radiological deterioration on repeat MRI scans (28.6%), followed by lateral recess discs (17.9%), as compared to central discs (12.7%). Notably, among the patients who required emergency surgery, one had radiological deterioration in a paracentral disc, and two had radiological deterioration in central discs.

Our findings are consistent with previous studies that have reported discrepancies between MRI findings and clinical symptoms. For instance, Carragee et al. conducted a study in which baseline MRI scans were performed on 200 patients who were followed up for five years, with repeat scans conducted on those who developed episodes of low back pain [7]. They found that 84% of patients who developed clinically worsened symptoms showed either unchanged or improved lumbosacral imaging findings, which aligns well with our findings of 85% of patients showing either unchanged or improved imaging [7]. Further to this, el Barzouhi et al. found that MRI scans conducted at one-year follow-up for patients who were conservatively treated for sciatica and lumbosacral disc disease did not reliably distinguish between those with favourable and unfavourable outcomes [8]. These studies collectively suggest that repeat lumbosacral MRI findings may not always correlate with patient symptoms and may not necessarily alter the management approach for most patients.

These findings underscore the importance of carefully considering the clinical context and evaluating the clinical symptoms in conjunction with radiological findings when interpreting repeat MRI scans in patients with lumbosacral degenerative disc disease, as changes in MRI may not always directly translate to changes in patient management [9].

The natural history of degenerative disc disease

The natural radiological progression of degenerative lumbosacral disc disease often involves improvement or regression of the disc with conservative management. Wang et al. conducted a meta-analysis of studies on non-surgically treated symptomatic lumbar disc herniation patients and found an overall disc regression rate of 63% on repeat MRI scans, indicating that a significant proportion of patients may experience improvement in disc disease over time [10].

Moreover, Chiu et al. performed a systematic review of studies that investigated disc regression based on the type of herniation and found higher rates of regression in disc sequestration (96%), followed by disc extrusion (70%), disc protrusion (41%), and disc bulging (13%) [11]. This suggests that different types of disc herniations may exhibit varying rates of regression, with disc sequestration showing the highest regression rate, potentially due to increased inflammatory response and breakdown of nucleus pulposus material in the spinal canal [12].

The timing of repeat MRI scans is also an important consideration. Several studies have shown that there may be no radiological change when an MRI is repeated within 60 days, regardless of symptoms [13,14]. Our results support this finding, as no patient demonstrated radiological improvement within the first 60 days after the initial scan. Additionally, we observed that none of the patients who demonstrated radiological deterioration within 60 days of the initial scan demonstrated "clinically significant" deterioration in the form of CES. The earliest clinically significant change requiring neurosurgical intervention appeared in the 61-90-day interval, suggesting that changes in MRI findings may become more evident beyond the initial 60-day period, a finding that is mirrored in the literature [13].

The burden of excessive scanning

Darlow et al. highlight the potential psychological effects of excessive MRI in patients with degenerative disc disease [15]. Patients' interpretation of imaging abnormalities, such as "bulge," "annular tear," or "herniation," which are often used interchangeably and interpreted differently by clinicians, can lead to beliefs that their spine is structurally vulnerable. This can trigger fear-avoidance behaviours and detrimental beliefs that may predispose patients to chronicity [16]. Furthermore, although reported changes on MRI scans would have resulted in no immediate change to management in most cases, repeating scans can increase the risk of chronicity and potentially result in worse clinical outcomes for patients [17]. Additionally, misinterpretation of MRI scans by clinicians may lead to unnecessary further investigations and increased healthcare costs [18].

In our experience, a significant proportion (16.6%) of repeated MRI scans within 12 months were due to patient factors such as anxiety, claustrophobia, and discomfort, resulting in abandoned or incomplete scans (130 out of 783 scans). This represents a sizable number of scans, and strategies should be implemented to anticipate and address such problems to minimise the waste of limited MRI scan opportunities [19]. This underscores the importance of considering patient factors and optimizing patient comfort during MRI to ensure efficient utilization of resources and provide optimal care to patients [20].

Limitation

One limitation of our study is the lack of longitudinal follow-up beyond the 12-month period. Longitudinal data would have provided insights into the durability of observed changes in radiological findings and their correlation with clinical outcomes over time.

## Conclusions

Our study offers valuable insights into the management of lumbosacral degenerative disc disease, particularly concerning the role of repeat MRI scans in altering patient management. The findings underscore the limited impact of repeat MRI scans within a 12-month period on altering clinical management, with the overwhelming majority (81%) of scans showing no significant change in radiological findings. Moreover, the incidence of clinically significant deterioration, as evidenced by the development of CES, is remarkably low (0.62%) in this cohort, highlighting the rarity of emergent cases within this timeframe.

Consistent with prior research, our study reaffirms the discordance between MRI findings and clinical symptoms, emphasizing the importance of contextualizing radiological assessments within the broader clinical picture. Despite advancements in imaging technology, repeat MRI findings may not always correlate with patient symptoms, necessitating a nuanced approach to clinical decision-making. Furthermore, the natural history of degenerative disc disease suggests a propensity for regression or improvement with conservative management, emphasizing the need for judicious interpretation of imaging findings.

In conclusion, our findings advocate for a balanced approach to utilizing repeat MRI scans in the management of lumbosacral degenerative disc disease, emphasizing the primacy of clinical judgment and the need for personalized, patient-centred care. Further research endeavours should focus on refining imaging protocols and elucidating the long-term implications of repeat imaging on patient outcomes.
